# How to Live Long

**Published:** 1889-11

**Authors:** S. H. Preston


### HOW TO LIVE LONG.

BY S. H. PRESTON.

The world is wearied with books on the preservation of health, and with learned lectures on the attainment of longevity. Very few of them are of the least practical value. The reader learns about lacteals, lumbar lymphatics, albumen, azotised substances, and lots of similar stuff, but when he gets through he likely knows no more about what Bill Nye calls his "Seth Thomas works" inside, or the course of living to secure health and prolong life than previous to their perusal.

It will be found that most of centenarians were hard working people in common life, and knew nothing of the constituent elements of the body, the chemistry of digestion, or of the osseus structure. Several of the most remarkable longest-livers were inmates of asylums or poor-houses. Their habits of life were as varied as their characters.

Some of the most inveterate tobacco users lived to be over one hundred years. A Prussian peasant died a few years ago at the age of 109, whose life was supposed to have been cut short by excessive smoking. The daily diet of a woman who lived to be 117 is said to have been buttermilk and greens. One old toper who reached 104 drank a pint and a half of London gin daily.

An Irishman who imbibed plentifully of rum and brandy lived to be 111. With some extraordinary exceptions like these, the majority of long-livers were characterized by correct hygienic habits. The exceptions show there is no reliable rule for the attainment of old age.

It is likely that a tendency to longevity is frequently inherited—that it runs in families. We know that a lack of vitality, weakness of constitution, and the tendency to certain diseases are transmissible. Premising that nature never indicates any physiological preference for individuals, we can account for this principle of preservation by the fact that in all large families the weakest and diseased die out, leaving only the healthy and most hardy to propagate. With such sound stock long life through successive generations is the result.

If the truth be plainly told, death is usually but a species of suicide. People seem set upon the very mode of life that will kill them quickest. Too much eating and too little sleeping, stimulants, excitement, and reckless dissipation, brains overburdened with business, hearts harrowed with the cares and responsibilities of life—such are some of the things that are taking people off. Worry and nervous excitement kill folks faster than hard work. Steady, honest, hard-handed labor never hurt anybody. The placid, patient, plodding person, other conditions being the same, lives the longest.

Much depends upon the conservation of the physical force in youth. The ancient physiologist pointed out the fact that in early life there is a great deal of this force in reserve, as a sort of stock to meet the demands of advancing years. With the increasing cares and strain of sterner duties in later life, this stock is correspondingly diminished. It becomes prematurely exhausted, and existence ends in bankruptcy. So that the proper way to prolong life is to make the body a sort of savings bank for this original stock of strength in youth and early manhood.

Nothing so soon destroys the vigor of life as excessive emotions. Worry will waste the most robust body, care will corrode, and even an excess of joy prove deadly. The wise will avoid everything that overtakes the feelings. The man who can continually maintain his equanimity has the best chance, all things considered, of living a long and happy life. So from a merely physiological point of view, we should exercise composure under all circumstances ; love nothing too passionately, hate nothing too violently, fear nothing too strongly, lament nothing too sorrowfully, and tranquilly and trustfully accept the order of things as we find them untroubled

> "With the time which drove
> O'er our content its strong necessities,
> And let determined things to destiny
> Hold unbewailed their way."

To a person who has properly preserved himself the period between 45 and 60 will be the prime of life. Experience has ripened his judgment, and matured strength of constitution will enable him to withstand an attack of disease. He has triumphed over the storms and struggles that threatened his early manhood, mastered his business, secured a competence and rest from wearing work, and safely crossed the viaduct called the "turn of life," which is usually an entrance upon a prolonged pilgrimage or a short turn to the tomb. He is now at his best, and all his faculties having attained their fullest expansion, either begin to gradually close like dahlies at the setting of the sun, or drop as though touched by a destructive frost. If he be spared, he can gird up his loins, get a stouter staff, and trudge on over the bridge that leads to old age.

There is no natural death but old age, which, if death may ever be called pleasant, is the only pleasant one. There is a wish for rest, and the tired traveler sinks to slumber in a silent valley at the close of a well-spent day.

